# Emotional reactions and psychological responses expressed by adolescent and adult women survivors of sexual violence during outpatient follow-up

**DOI:** 10.61622/rbgo/2025rbgo37

**Published:** 2025-07-15

**Authors:** Ana Luiza Teixeira, Stephanie Oliveira de Lima, Daniela de Oliveira Godoi, Alejandra Suyapa Becerra-Torres, José Paulo Guida, Renata Cruz Azevedo, Arlete Fernandes

**Affiliations:** 1 Universidade Estadual de Campinas School of Medical Sciences Department of Obstetrics and Gynecology Campinas SP Brazil Gynecological Division, Department of Obstetrics and Gynecology, School of Medical Sciences, Universidade Estadual de Campinas, Campinas, SP, Brazil.; 2 Universidade Estadual de Campinas School of Medical Sciences Department of Medical Psychology and Psychiatry Campinas SP Brazil Department of Medical Psychology and Psychiatry, School of Medical Sciences, Universidade Estadual de Campinas, Campinas, SP, Brazil.

**Keywords:** Sexual violence, Sex offense, Psychological responses, Women, Adolescent

## Abstract

**Objective:**

To evaluate psychological support data for survivors of sexual violence (SV) and compare the attitudes, responses, and feelings in adolescent and adult women.

**Methods:**

This was a retrospective study with two cohorts of female survivors of sexual violence, treated between 2011 and 2022. Women who had at least one psychological evaluation were included. The variables were sociodemographic; characteristics of violence; feelings; attitudes; symptoms observed/reported during support; time until emergency care; and indication of psychotropic medications. We calculated the mean and standard deviation (SD) and used the λ-Square or Fisher's Exact test and the Mann-Whitney test for comparative analysis. The significance level adopted was 5%.

**Results:**

Five hundred and twenty-one adolescents, mean age 14.8 (SD±2.0) and 312 adult women, mean age 31.7 years (SD±10.7), were compared. Two-thirds of all women reported themselves as white; adolescents took longer to seek care (p<0.001) more frequently than the adult group. Adult women had more histories of sexual abuse (p<0.001), penetration attacks (p<0.001), reported greater perception and disclosed violence more frequently (p<0.001) than the adolescent group. Adolescents reported more shame (p<0.001) while the group of adults more frequently expressed feelings of insecurity, anguish, expressions of crying, revolt, anger, humiliation and apathy. Anxious symptoms were expressed by 60% of adults and 44% of adolescents and the prescription of psychotropic medications was higher in the adult group compared to adolescents (p<0.001).

**Conclusion:**

Both groups of survivors suffered psychological impacts after SV, expressing/reporting different reactions to distress. These results highlight the importance of access to psychological support after SV.

## Introduction

Globally, 31% [uncertainty interval (95%); UI 27–36%] of women aged 15–49 have been subjected to physical and/or sexual violence from any current or former husband or male intimate partner, or to sexual violence from someone who is not a current or former husband or intimate partner (IPV), or to both these forms of violence at least once since the age of 15.^(1)^ If added to the numbers of child abuse carried out by different perpetrators and suffered by girls aged <15 years, this prevalence would be higher.

In addition to constituting a public health problem and violation of human rights, violence against women has also been considered a public mental health problem due to its association with an increased risk of mental disorder.^(2)^ A review study evaluated the different forms of violence against women and girls and described repetitive violence, severity of violence and more recent violence as being associated with greater mental health morbidity.^(3)^ Negative effects of SV on general health and quality of life have been described, specifically affecting the psychological well-being and sexuality of women.^(4)^ Exposure to trauma in adolescence, including SV, can lead to several negative consequences on mental health, with a greater risk of difficulties in social adjustment and family functioning.^(5,6)^

A meta-analysis included 32 cross-sectional studies published from 2001 to 2022, with 19,125 participants, and described that 29% of women worldwide were victims of sexual violence, more than half developed post-traumatic stress disorder (PTSD) after abuse, and only a third of them considered seeking support.^(7)^ These results showed that women can experience various effects and psychological damage after SV and highlight the need for accessible fostering services and mental health support.

While the initial approach to minimizing harm to mental health is important, few studies have followed survivors after the attack regarding the persistence and recovery of symptoms.^(8,9)^ A systematic review sought to assess which psychosocial interventions work for victims/survivors of recent sexual assault; with ten studies and including range of interventions.^(10)^ The study concluded that the evidence is sparse and scientifically weak, and that there is a gap in the evidence base on psychosocial interventions for victims/survivors of sexual assault.^(10)^

Due to so many injuries and the lack of comparative studies on how sexual violence impacts women of different ages, we chose to evaluate the data found during psychological support in a reference service for survivors of SV, and compare attitudes, psychological responses and feelings triggered in adolescent and adult women. It is possible that we better understand the psychological responses expressed and/or reported in different age groups, we can create different care approaches with implementation in the quality of care.

## Methods

This was a retrospective study. Data from two cohorts of female survivors of sexual violence treated at a university hospital were compared. For this study, the REC was asked to waive the Informed Consent Form, which was accepted. We followed all items in the strengthening of the communication of observational studies in epidemiology (STROBE).

The study setting is a reference for tertiary health care in the metropolitan region of Campinas, São Paulo, Brazil, with coverage for an urban population of about 4.0 million inhabitants. This university hospital has been a reference in the Unified Health System (UHS) in providing care to victims of sexual violence since 1998. Health care is carried out by a multidisciplinary team and follows public health policy protocols.^(11)^

A database was created for the current research that included two cohorts of women. The first cohort included adult female (≥20 years) survivors of SV who received emergency care from 2014-2022. The second cohort included data from previously published research conducted with adolescent female survivors of SV who received emergency care from 2011-2018.^(12)^ Both cohorts had data collected from medical records stored in the hospital database.

The service flow is the same for adolescent and adult women and follows the service protocol.^(13)^ Reception and emergency care are provided by a nurse and a doctor on duty; when necessary, the mental health team is called. After emergency care, psychological evaluation is carried out in outpatient care and follow-up is offered for six months. Adolescents also receive support and guidance from family members/legal guardians.

The psychology team is made up of two hired psychologists with experience in the area and a group of psychologists in a professional development program and/or in-service training, who receive guidance on completing a specific psychological form. At each outpatient follow-up, the psychology team uses this form to systematically collect information on feelings, symptoms, reactions and attitudes expressed and/or related. All women whose data were included in this analysis received at least one assessment from the psychology team.

The variables studied were sociodemographic data (age, self-reported skin color, schooling years and religion); type of abuse (acute = an event that is not repeated; chronic abuse = when the aggression is repeated over time and is perpetrated by the same aggressor/aggressors); whether aggression was facilitated through social media (social network, uber, etc.); characteristics of violence [if there was intimidation, known aggressor or not, type of violence (vaginal, oral, anal aggression)], personal history of sexual abuse; feelings (shame, guilt, crying, humiliation, apathy, anguish, rage, revolt, insecurity); attitudes after SV [SV perception, disclosure about SV, changes in daily routine, address changes, social isolation], reactions/symptoms observed/referred during outpatient follow-up for mental disorders (anxious symptoms, sleep disorder, suicidal behaviors, flashbacks, fear of the SV consequences); indication for psychotropic medications during follow-up; and time until emergency care (≤24 hours, >24-72 hours, 72 hours-5 days, >5 days-6 months and > 6 months). Skin color was self-reported as black, brown, white, yellow or indigenous.^(14)^ In this study, we categorized self-reported skin color as white and non-white (black, brown, yellow and indigenous).

The analysis was descriptive with absolute frequency and percentage values for categorical variables and mean values, standard deviation (SD), minimum and maximum values, median and quartiles for numerical variables. We used the λ-Square or Fisher's Exact tests to compare categorical variables and the Mann-Whitney test to compare numerical variables. The significance level was 5%. We used the Statistical Analysis System for Windows, version 9.2 (SAS Institute Inc, 2002-2008, Cary, NC, USA).

The study was approved by the institutional Research Ethics Committee 4.730.364 (*Certificado de Apresentação de Apreciação Ética:* 45387521.0.0000.5404).

## Results

**Figure 1:**
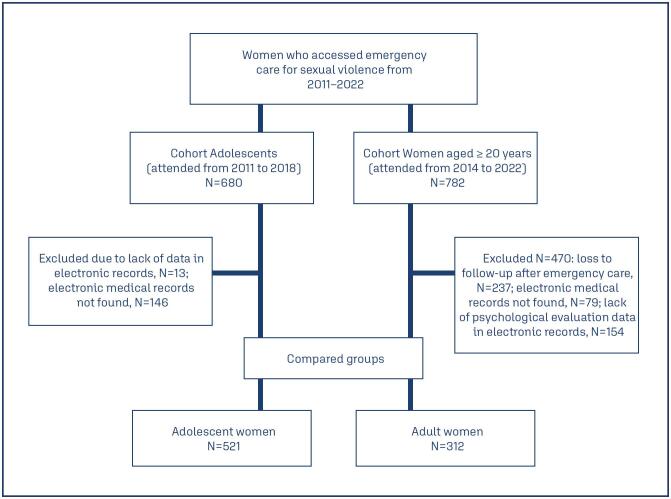
Flowchart of the comparison groups analyzed in the two surveys

The comparison groups consisted of 521 adolescents (10 to 19 years old), mean age of 14.8 years (SD±2.0; 10 to 19 years), attended from 2011 to 2018; and 312 adult women, mean age of 31.7 years (SD±10.7; 20 to 74 years) attended from 2014 to 2022 (Figure 1).

In the total sample of women, 246 (33.3%) declared themselves non-white (Table 1). We found a higher number of adolescents who declared themselves non-white when compared to the group of adults (p=0.019); however, there were no differences between the groups after adjusting for years of schooling (p=0.443). Adult women had a higher level of schooling when compared to adolescents (p<0.001) (Table 1). We found a higher frequency of adolescents of Protestant religion, of evangelical tradition, when compared to adults; the result was maintained after adjusting for years of schooling (p<0.001) (Table 1).

**Table 1:** Characteristics of women victims of sexual violence according to age group

Sociodemographic characteristics		Age groups		p-value*	p-value**
		10-19 years	≥20 years		
		n=521	n=312		
Self-reported skin color (n=822)				0.019	0.443
	White	332(63.7)	216(71.7)		
	Non-White	189(36.2)	85(28.2)		
Schooling years (n=808)				<0.001	-
	≤ 8	260(50.3)	39(13.4)		
	≥ 9	257(49.7)	252(86.6)		
Religion (n=480)				<0.001	<0.001
	Protestant-Evangelical tradition	199(41.4)	77(27.2)		
	Catholic	149(31.0)	90(31.8)		
	Others	28(5.8)	33(11.6)		
	No religion	104(21.6)	83(29.3)		

* Chi-square Test.

** Adjusted for Schooling years, Multiple logistic regression analysis; analyzed variables: education, self-reported skin color and religion

Acute abuse was the most prevalent in both groups, with no differences, and less than 5% of the sample of women had a history of chronic abuse (Table 2). Both groups had a similar occurrence of a known aggressor, intimidation and the facilitation factor of some social media. Adult women had a higher frequency of vaginal, anal and oral aggression when compared to the group of adolescents (p<0.001). The number of adults who arrived for emergency care was higher in the first 24 hours, compared to adolescents who arrived more frequently at the emergency room after 72 hours until the 6th month (<0.001). The history of sexual abuse was also higher among adults compared to adolescents (p<0.001) (Table 2).

**Table 2:** Characteristics of sexual violence and emergency care according to age group

Characteristics of sexual violence and emergency care		Age groups		p-value*
		10-19 years	≥20 years	
		n=521	n=312	
Type of abuse (n=809)				
	Acute	493(94.6)	277(96.1)	0.323
	Chronic	28(5.3)	11(3.8)	
Known aggressor		279(53.6)	136(49.8)	0.304
Social media – facilitating factor (n= 807)		19(3.6)	17(5.8)	0.149
There was intimidation (n=491)		418(85.1)	206(86.2)	0.703
Vaginal aggression		331(63.5)	210(88.6)	<0.001
Oral aggression		83(15.9)	82(34.6)	<0.001
Anal aggression		76(14.6)	83(35.0)	<0.001
Emergency Care				
Time until medical care (n=815)				<0.001
	≤ 24 h	262(50.9)	193(64.1)	
	> 24–72 h	98(19.0)	58(19.2)	
	> 72 h–5 days	42(8.1)	9(3.0)	
	> 5 days until 6 months	107(20.8)	38(12.6)	
	> 6 months	5(0.9)	3(1.0)	
History of sexual abuse (n=651)		87(17.0)	75(53.5)	<0.001

* Chi-square test

Regarding feelings, the group of adolescents reported more shame (p<0.001) while the group of adults more frequently expressed feelings of insecurity, anguish, expressions of crying, revolt, anger, humiliation and apathy (p<0.001) (Table 3). Most women in both groups reported having a perception of the violence they suffered, but the adult group had a greater number of women with this attitude when compared to adolescents (p<0.001). The same occurred for disclosure attitudes about VS (p<0.001); changes in daily routine (p<0.001) and change of address (p=0.042) more mentioned by the adult group when compared to the adolescent group (Table 3). Adolescents reported sleep disorders more frequently than adults (p<0.001). Anxious symptoms were expressed by 60% of adults and 44% of adolescents (p<0.001) and the prescription of psychotropic drugs was higher in the adult group compared to adolescents (p<0.001) (Table 3).

**Table 3:** Feelings, attitudes, symptoms of mental disorders and indication of psychotropic medications during outpatient follow-up according to age group

Variables	Age groups		p-value*
	10-19 years	≥20 years	
	n=521	n=312	
Feelings (n=675)			
Shame	208(57.1)	114(36.6)	<0.001
Guilt	159(43.6)	113(36.3)	0.052
Crying	37(10.1)	130(41.8)	<0.001
Humiliation	24(6.6)	80(25.7)	<0.001
Apathy	18(4.9)	67(21.6)	<0.001
Anguish	14(3.8)	175(56.2)	<0.001
Rage	9(2.4)	93(29.9)	<0.001
Revolt	11(3.0)	95(30.5)	<0.001
Insecurity	11(3.0)	184(59.1)	<0.001
Attitudes			
Sexual violence perception (n=780)	465(93.3)	279(98.9)	<0.001
Disclosure about the sexual violence (n=575)	280(67.6)	151(93.8)	<0.001
Changes in daily routine (n=454)	75(20.6)	84(93.3)	<0.001
Changed address (n=670)	31(8.5)	41(13.4)	0.042
Social isolation (n=670)	93(25.5)	81 (26.4)	0.787
Symptoms of mental disorders			
Anxious symptoms (n=675)	160(43.9)	188(60.4)	<0.001
Sleep disorder (n=675)	151(41.4)	87(27.9)	<0.001
Suicidal behavior (n=675)	31(8.5)	26(8.3)	0.942
Fear of the SV consequences (n=674)	47(12.9)	29(9.3)	0.146
Flashbacks (n=675)	65(17.8)	42(13.5)	0.123
Psychotropic prescription (n=641)	112(30.2)	155(57.4)	<0.001

* Chi-square test.

SV: Sexual Violence

## Discussion

Although psychological care is offered equally to adolescent and adult women, with psychotherapeutic support available for six months, it is important that the service be able to accommodate individual demands and, whenever possible, identify differences in patient subgroups.

This study found that adult women showed more frequent psychological responses when compared to adolescents. Specifically, anxiety symptoms were more prevalent in adults, for whom there was a greater need for the use of psychotropic medications during follow-up. The result of greater psychological suffering in adults was highlighted by the more frequent history of abuse in this group and whose justification was that the longer life span provided greater exposure. Violence against women is common and specifically SV occurs in different environments and moments of life, from childhood to old age. National data showed the different types of violence suffered by female victims in the year 2022.^(15)^ Specifically for SV, the prevalence described was 17.9% among victims aged 0 to 9 years, 25.2% among those aged 10 to 14 years, ranged from 30 to 34% of occurrences among women aged 15 to 64 years and decreased progressively from 26.8% in the age groups over 65 years to 21.9% in female victims aged ≥80 years.^(15)^

SV covers any sexual act without consent, attempted or committed against a person, and also the act perpetrated against a person incapable of consenting, whether temporarily or permanently.^(16)^ For the adult woman, the lack of consent was perceived more clearly. On the contrary, SV suffered by children and adolescents is often described as undetermined, due to the victim being unable to characterize the situation suffered. In children and adolescents, SV can occur in different ways, with physical and psychological violence, coercion, unwanted sexual contact by acquaintances, harassment, rape during dating and frustrated attempts at penetration.^(4,17)^ Proximity to the aggressor and fear of reprisals can also inhibit the perception of SV or its disclosure.^(4,17)^

In 5% of all women, SV was chronic or repetitive, a factor described as associated with greater mental health morbidity.^(3)^ As this service aims to provide emergency care with a focus on prophylactic treatment in the first 72 hours and the gateway to psychological support is the acute episode of SV, chronic/recurrent abuse is characterized during care in fewer women.

Adolescents took longer to seek emergency care, compared to adults, a result in agreement with other services around the world.^(4,6)^ The biggest implications of this result are clinical, with less indication of use of prophylaxis such as emergency contraception and STIs for adolescents. The delay in seeking care reflects the difficulty in telling someone you trust about the sexual assault. In this study 1:3 adolescents had not disclosed SV to anyone at the time of emergency care. One study sought to identify factors that can facilitate or inhibit child sexual abuse disclosures and described that younger age and male sex are strong predictors of delayed disclosure or withheld disclosure; which is also influenced by the proximity of the perpetrator, family member or acquaintance.^(18)^

We found that feelings of shame and guilt were reported more by adolescents when compared to adults, a result in agreement with a national study carried out with 205 victims of SV aged 6-14 years, 130 of them female, and which described that 2:3 girls reported feelings of guilt and shame.^(19)^ Feelings of shame, self-blame and fear are described as psychological barriers to disclosing SV,^(18)^ reinforcing the impact of the cultural environment and promoting negative outcomes related to mental health.

A perspective article published by Australian authors presented a broad discussion of SV in rural/remote areas and the association with shame discourses that are used as an informal mechanism of social control for women, but also cause pain, exposure, guilt and humiliation, embarrassing victims and contributing to the increase in adverse outcomes of aggression and trauma.^(20)^ We observed more than 40% of adolescents with anxious symptoms and sleep disorders, a very high prevalence that denotes the trauma experienced. Exposure to trauma in adolescence has been described as a factor that can lead to several negative consequences on mental health,^(6)^ and exposed adolescents are more likely to suffer sexual abuse in adulthood.^(4)^

The feelings most mentioned by the adult women in this study were similar to those described in the "rape syndrome" described in 1974 in a study with 92 adult female rape victims hospitalized during a one-year period.^(21)^ The authors described that after acute trauma women experienced feelings of shock and disbelief, confusion, mood swings, disgust, humiliation, shame, self-blame, helplessness, anger, revenge, desire to forget and inability to talk about and describe the violence. In the next phase, called reorganization, women experienced feelings of insecurity, dependence or lack of autonomy, loss of self-confidence and self-esteem.^(21)^ Currently, studies and reviews have described that different populations of women experience different forms of violence throughout their lives and express different degrees of mental disorders in the face of trauma.^(5,6,22-24)^ These studies have described responses of anxiety, depression, phobias, attempts at self-harm, suicidal ideation, drug use, eating disorders, sleep disorders and psychosomatics; often describe having nightmares, recurring thoughts, flashbacks, reduced attention/concentration or hypervigilance; they may still have difficulties with their sexuality and interpersonal relationships.^(5,6,22-24)^

Regarding age group, the specialty guideline highlights the importance of family support when it comes to adolescents.^(25)^ Psychological support should be provided to all victims of sexual violence and aims to strengthen self-esteem, restore protection and coexistence in dignified living conditions, contributing to overcoming the situation of rights violation and to reparation for the violence suffered.^(25)^ Within the community, networking for social, medical, legal, psychological and pedagogical interventions is essential to organize the recovery of survivors of all ages. Particularly for adolescents these protective actions by the psychologist and the care service, including family members whenever possible, are important to prevent the repetition of violence.

We found that two-thirds of the total sample of women (66.6%) self-reported as white, which contradicts national data on the Brazilian population that describe that 43% self-declare as white and 56% brown or black skin color.^(14)^ It is possible that there were errors in the collection of skin color data or that the information was not self-reported but recorded by a professional inserted in a context of institutional and structural racism; but it is also possible that women belonging to the lowest socioeconomic stratum and with greater vulnerability to suffering SV did not have access to this assistance service. They may not have information about the existence of services, about the importance of emergency care after SV, or have difficulty reaching the service.

This study had limitations in its retrospective design, with loss of information in some variables. Other limitations were the different temporal cohorts, which may also have contributed to bias in the results. However, we consider that the common sequential period was longer, from 2014 to 2018 and included both groups, which may have minimized possible errors. Another limitation may have been the collection of some data systematically during psychological care in our service. For some analyses, systematic collection can increase reliability by encompassing all services, and on the other hand, it can lead to errors due to the lack of depth of information.

On the other hand, we consider that the strength of this study was being able to compare some aspects of psychological suffering between adults and adolescents. It has always been a perception of professionals who provide assistance to survivors during outpatient follow-up, that the population of adult women expresses suffering more frequently, and of greater intensity, when compared to adolescents. Prospective studies must be carried out to better evaluate the psychological responses triggered at different ages.

## Conclusion

The results of this study indicate that adolescents verbalize and demonstrate their suffering differently than adults, in particular, with higher rates of feelings related to the social impact of SV, reporting more frequently shame and feelings of guilt, which should be worked on in psychotherapeutic care; adult women expressed greater distress, but both groups of survivors suffered different psychological impacts after SV. These results highlight the importance of access to psychological support for women who experience SV at any age, to detect alarm situations and provide opportunities for recovery and overcoming trauma.
